# Terrestrial Mammal Occupancy in the Context of Widespread Forest Loss and a Proposed Interoceanic Canal in Nicaragua's Decreasingly Remote South Caribbean Region

**DOI:** 10.1371/journal.pone.0151372

**Published:** 2016-03-23

**Authors:** Christopher A. Jordan, Cody J. Schank, Gerald R. Urquhart, Armando J. Dans

**Affiliations:** 1 Global Wildlife Conservation, Austin, Texas, United States of America; 2 Panthera, New York, New York, United States of America; 3 Department of Geography and the Environment, University of Texas at Austin, Austin, Texas, United States of America; 4 Department of Fisheries and Wildlife, Michigan State University, East Lansing, Michigan, United States of America; 5 Lyman Briggs College, Michigan State University, East Lansing, Michigan, United States of America; University of Colorado, UNITED STATES

## Abstract

Central America is experiencing rapid forest loss and habitat degradation both inside and outside of protected areas. Despite increasing deforestation, the Caribbean region of Nicaragua plays an important role in the survival or extinction of large mammal populations in Central America given that it still retains core areas of habitat for large mammal species. The proposed interoceanic canal project that would bisect the southern half of this Caribbean region represents a new threat that, combined with an advancing agricultural frontier, could affect populations of large mammal species such as jaguars, white-lipped peccaries, and Baird’s tapirs. We used occupancy models to examine the relative occupancy probabilities for an assemblage of terrestrial mammals in the south Caribbean region of Nicaragua to identify current core areas for our study species and conduct a preliminary evaluation of the potential impacts of the proposed interoceanic canal. We modeled a community level distribution of eight species with varying levels of sensitivity to human encroachment and a range of habitat associations. Our model results reveal three priority areas for terrestrial mammal conservation in our study area. The mapped predictions show that the only remaining area of suitable habitat for large mammals in the path of the proposed interoceanic canal is a relatively thin strip of forest that runs along the Caribbean Coast. In light of these findings, we propose five recommendations that will help ensure the conservation of this area of the proposed canal route as suitable habitat for our study species.

## Introduction

During the past fifteen years, Central America has rapidly emerged as one of the most environmentally threatened regions globally, with several countries posting some of the highest rates of forest loss in the world [1]. Indeed, Central America, a region that boasts Nicaragua as its largest country at 130,000 km^2^, has lost nearly 170,000 km^2^ of forest since 2000 [1]. Biologists and politicians collaborated in the early 2000s to design and protect a functioning biological corridor connecting the larger protected areas throughout the Central American isthmus. However, both the corridor as a whole and protected areas across all levels of protected status (i.e. Biosphere Reserves, Nature Reserves, Wildlife Reserves, etc.) have been degraded and/or destroyed by agricultural encroachment and unsustainable land use practices in recent years [2–4]. This forest loss has almost certainly resulted in precipitous declines in biodiversity generally and the isolation of many subpopulations of terrestrial mammals in increasingly small habitat patches and protected areas. It is essential to identify remaining core areas for key mammal species in this region and begin to bolster their protection.

**Fig 1:**
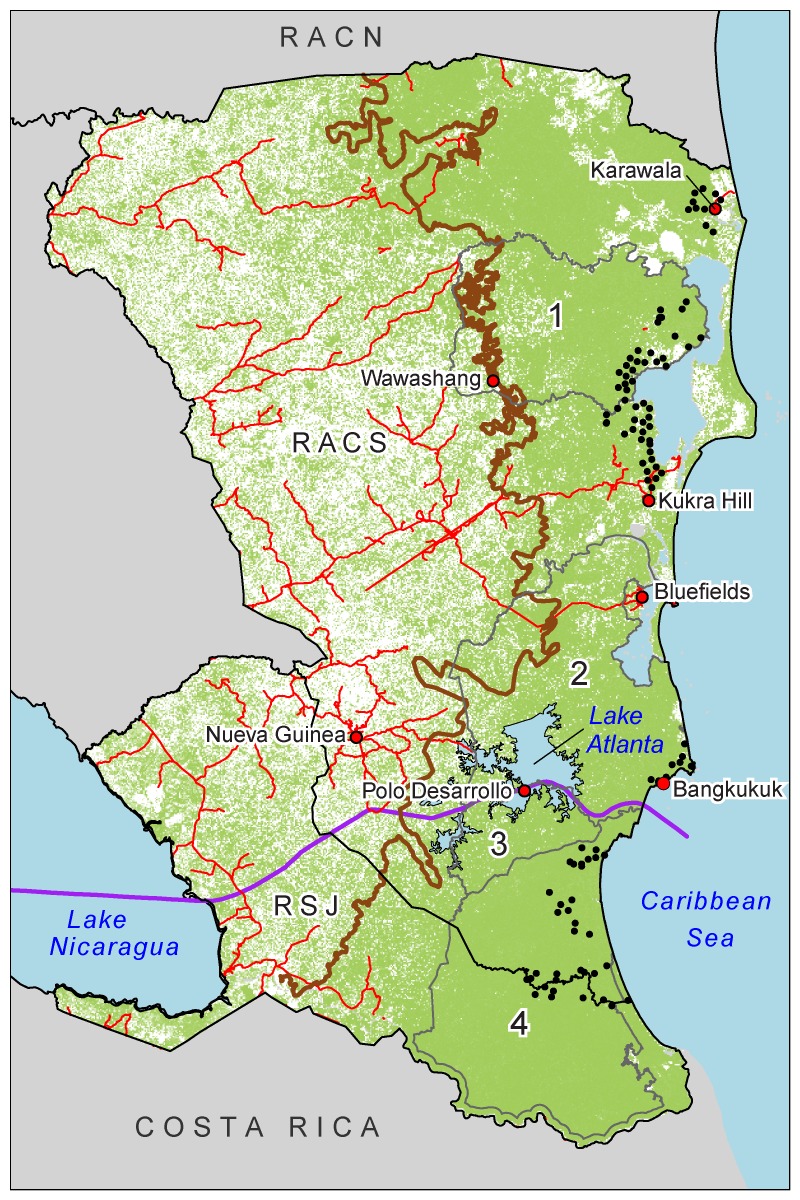
Map of southeastern Nicaragua illustrating the proposed canal route (purple line) and Lake Atlanta (light blue), the agricultural frontier as interpreted from the 1999–2001 land cover map (brown line), forest cover (green), camera trapping locations (black circles), major roads (red lines) and Department boundaries (black outline), in the Southern Caribbean Autonomous Region and Rio San Juan province. Most relevant protected areas for this paper (outlined in grey) are numbered: 1) Cerro Wawashang, 2) Cerro Silva, 3) Punta Gorda, and 4) Indio Maiz.

The Caribbean region of Nicaragua, more specifically, the South Caribbean Autonomous Region (RACS), along with its two neighboring departments of Rio San Juan (RSJ) and the North Caribbean Autonomous Region (RACN), are believed to harbor some of the best habitat for terrestrial mammals in Central America (**Fig 1**) [5]. The RACS, RACN, and RSJ comprise over 50% of Nicaragua’s national territory, encompass the entire Caribbean border of Nicaragua from Honduras to Costa Rica, and are the most forested regions remaining in the country. The core areas of Nicaragua’s two biggest reserves, the Indio-Maíz Biological Reserve and the Bosawás Biosphere Reserve, are primarily within the RACS, RACN, and RSJ. Even recently the joint forested ecosystems of the RACS, RACN, and RSJ were assumed to serve as an active genetic corridor for the globally endangered Baird's tapir [6] and most likely remain a viable corridor between Honduras and Costa Rica for jaguars and pumas [7]. Given this, and that Nicaragua is the largest country in Central America, we posit that the natural areas in the RACS, RACN, and RSJ will play a key role in the survival or the extinction of large terrestrial mammal populations in Central America in the coming decades. Thus we believe more detailed analyses of the terrestrial mammals of these regions, their current threats, and potential strategies for protecting conservation priority mammal species and their remaining core areas are needed.

In this paper, we offer a case study from research conducted in the RACS and RSJ. A culturally diverse set of human communities from six main ethnic groups occupies and relies on RACS and RSJ ecosystems: the Rama, Miskito, and Ulwa indigenous people, the Garifuna and Kriol Afro-descendant groups, and colonist *Mestizo* cattle ranchers who recently migrated from Nicaragua’s dry Pacific region. The first five groups communally own and historically managed the overwhelming majority of local lands, using local forests for hunting and clearing small tracts of land for subsistence, swidden agriculture. Under laws 445 and 28, these indigenous and Afro-descendant groups have legal, communal tenure to all lands where we conducted fieldwork. In the last four decades, an agricultural frontier of colonist *Mestizo* cattle ranchers has advanced into the Caribbean lowlands, clearing forests and establishing cattle ranches illegally within the poorly monitored areas of the indigenous territories. Research indicates that the Afro-descendant and indigenous land-use and hunting practices are more sustainable than those of the colonist *Mestizo* cattle ranchers [8]. The population of colonist *Mestizos* in the RACS is now larger than the indigenous and afro-descendant population, and the associated cattle ranching frontier has become the primary environmental threat in the region. If not addressed, this frontier alone may effectively eliminate all remaining protected areas in Caribbean Nicaragua within the next 10–20 years; it has already severely degraded or destroyed three nature reserves since the year 2000 [3–4].

In this already environmentally precarious context, the Nicaraguan National Assembly passed Law 840 in June 2013, granting a concession of an undefined tract of land in Nicaragua to the Hong Kong Nicaraguan Canal Development Group (HKND) to construct an interoceanic canal [9]. In addition to the canal and its related infrastructure, the law grants HKND the rights to implement a variety of development projects along the canal route, including international airports, tourist complexes, an oil pipeline, free-trade zones, and two deep-water ports (**Fig 1**). The canal itself will traverse 272 km of Nicaragua, including 105 km through Lake Nicaragua. The ecosystems of the Caribbean Region will be affected by a deep-water port in Bangkukuk, the 395 km^2^ artificial Lake Atlanta, the Caribbean Coast Camilo Locks, the Punta Aguila Port Access Road [10], and probably by ancillary development projects. The rapid progress has generated great concern about the potential impacts of the canal and its supporting infrastructure on the environment in Nicaragua [9].

To begin examining how increased *Mestizo* encroachment and the proposed interoceanic canal may affect terrestrial mammals and their core areas of habitat in the RACS and RSJ, we examined the occupancy rates of a terrestrial mammal assemblage in the area’s remaining forested landscapes, and projected the results onto species-specific occupancy maps. We used results to identify remaining large tracts of highly suitable habitat for large mammals, assess the effects of different threats to the continued occupancy of terrestrial mammals throughout the entire study area, and observed the relative rates of occupancy for our study species in the vicinity of the proposed canal related infrastructure to assess the potential implications of this large development project.

## Materials and Methods

### Study Area

We conducted our research primarily in the RACS, Nicaragua, and conducted brief surveys in 2014 in the neighboring department of RSJ (**Fig 1**). The RACS and RSJ comprise a highly biodiverse region due to wide variations in soil composition, topography, and elevation [11]. Its most common tropical ecosystems include mangrove forests, lowland tropical rainforests, seasonally flooded palm forests, permanently inundated *Raphia taedigera* Mart. palm swamps, and pine savannahs. Combined, the RACS and RSJ have seven protected areas of appreciable size, including the Indio-Maíz Biological Reserve, The Cerro Wawashan Nature Reserve, the Cerro Silva Nature Reserve, the Punta Gorda Nature Reserve, the Llanos de Karawala Reserve, the San Juanillo Wildlife Refuge, and the Río San Juan Wildlife Refuge (**Fig 1**).

### Camera-trapping

We conducted camera trapping in the RACS and RSJ (N 11.04293 to 12.97717, W -84.05253 to -83.56387) during 2010, 2011, 2012 and 2014 for two simultaneous wildlife research projects (**Fig 1**). We conducted the 2010, 2011, 2012 surveys and part of the 2014 surveys to assess terrestrial mammal occupancy in the RACS (detailed in Chapter Four of [12]). Specifically, we placed a grid of 4 km^2^ cells over the forests behind fourteen human communities within the study area. We randomly numbered these cells and then, starting with the lowest numbered cells, surveyed as many cells as possible around each community. The specific number per community varied from 2–8; this number was limited by the number of camera traps available for sampling (i.e. we did not have enough equipment to sample 30 cells per community), then determined by the extension of natural habitat around that community and the number of inaccessible cells near each community (we were unable to install camera traps in large wetlands, for example). We mandated a minimum distance between any two cameras of 2 km; this was maintained for all cameras across all years, meaning that each independent camera location was not within 2 km of any other independent camera location sampled at any time during this study. To choose specific camera locations within each cell, we hired local guides with intimate knowledge about local forests and asked them to lead us to specific locations known for high species richness. We installed cameras on game trails in one such location in each cell after assessing if all other methodological requirements were met. Thus sampling was random at the site level, but specific location of camera installation was determined by local knowledge and the presence of game trails.

We implemented a similar methodology for the remaining 2014 surveys. The key differences in methodology were that: 1) we used the same randomly numbered grid-based approach albeit with 36 km^2^ cells; 2) we asked knowledgeable local guides to lead us to locations where tapirs (17 cameras) or jaguars (1 camera) were known to frequent rather than locations with high species richness and installed cameras on game trails in these locations of “high tapir or jaguar traffic” (described in Chapter Two of [12]); and 3) for the 17 cameras set on game trails in areas of “high tapir traffic”, we mandated a minimum distance between adjacent cameras of 1.5 km.

We decided to merge data from across studies into the analyses in this paper after: 1) we calculated and compared the mean minimum distance between two adjacent cameras for each methodology and determined that it was not significantly different between studies (2.2 km for cameras placed for species richness, 2.4 km for other 2014 cameras); and 2) we tested for relationships between sampling method and detection probability for all of our study species and found no evidence of a significant relationship for any species.

All camera trapping fieldwork was conducted with an approved exemption from the Michigan State University Institutional Animal Care and Use Committee.

### Occupancy models

We used the basic hierarchical modeling framework described by Royle and Dorazio [13] and adapted code by Zipkin & Royle [14] and Linden [15] to estimate occupancy dynamics of an assemblage of medium to large sized mammals across the study site. This model estimates species-specific model parameters as random effects of a community-level distribution, which permits more accurate parameter estimates for rarely detected species [15]. We decided to use this particular model for our Nicaragua data because we had sparse detections of large mammal species across all years, and this model and others similar to it are frequently cited as being apt for accommodating such data.

We created species-specific detection histories by organizing the photos from the first 44 days of data from each camera by species and then dividing the species-specific data into 11-day sampling occasions. Some cameras malfunctioned prior to reaching the 44 day benchmark, in which case we used data only from all complete 11 days occasions captured by the camera. Each species’ detection history therefore consists of a string of two to four 1’s or 0’s, each indicating whether species *i* was detected (y = 1) or not (y = 0) during the 11 day sampling interval *k* at site *j*. We selected an 11-day sampling occasion due to prior experience observing Baird’s tapirs within the study area; tapirs appear to cycle through their entire home range over the course of 11–12 days [12].

The detections of rare species were not sufficient to allow us to run dynamic models across years with acceptable precision, thus we stacked data from all years into a single data sheet and included year as a fixed effect. Each of the study species thus had a unique detection history for each unique year/site combination. We chose species for our terrestrial mammal assemblage that had a range of sensitivities to human presence and ecological requirements in order to create a collective indicator of terrestrial mammal occupancy in the study region. In our final models, we included data on Central American agouti (*Dasyprocta punctata*), ocelot (*Leopardus pardalis*), lowland paca (*Cuniculus paca*), white-nosed coati (*Nasua narica*), white-tailed deer (*Odocoileus virginianus*), jaguar (*Panthera onca*), Baird’s tapir (*Tapirus bairdii*), and white-lipped peccary (*Tayassu peccari*). We consider the last three species to be our “rare species” group and the most sensitive to human encroachment. Jaguars are considered near threatened globally and critically endangered nationally [16]. Tapirs are considered endangered both globally and nationally [5–6,16]. White-lipped peccaries are classified as vulnerable globally, and although they were not evaluated for the Nicaraguan Red List, we consider them to be critically endangered in Nicaragua [17]. We consider ocelots and lowland pacas to represent species of intermediate sensitivity to forest degradation and human encroachment. We consider white-nosed coatis and white tailed deer to be least affected by alteration of primary forest habitat. We included agoutis as a generalist terrestrial mammal species.

We used logit-linear models for the probability of detection p_ijk_ and occupancy ψ_ij_ to model the effects of habitat covariates [15]. With one exception discussed below, we assumed that detection probabilities when species were present did not vary, and thus we defined the detection probability model as:

$$logit\left(p_{ij}\right)=\;\alpha_{i}$$

We produced a 1-km resolution grid for the study area, and used this as a snap grid for the environmental predictors used in modeling occupancy probabilities. We used eight total environmental predictors, in addition to modeling the fixed effect for year (YEAR). These included: 1) Distance to a significant road (ROAD) [18], 2) Distance to/within a protected area (DTPA) [19], 3) Area of swamps within 1 km buffer around camera site (SWAMP) [20], 4) Number of indigenous and afro-descendant communities within 10 km buffer around camera site (INDIG) [21], 5) Forest loss between 2000–2014 within a 1 km buffer around camera site (FORESTLOSS) [1], 6) Average forest cover within 1 km buffer around camera site (FOREST) [1], 7) Number of fires within 1 km buffer of camera site (FIRE) [22], and 8) A binary covariate indicating whether or not the site was within the Indio-Maíz Reserve (IM).

Given that forest loss is known to be higher around *Mestizo* settlements, we used FORESTLOSS as a proxy for *Mestizo* colonist encroachment. As mentioned above, *Mestizos* are primarily dedicated to cattle ranching, and this land use is the cause of essentially all net deforestation in the Caribbean region of Nicaragua [3,4], We compiled as much point data on *Mestizo* settlements as possible, but were not convinced that the resulting layer was consistently up to date across the study area and thus decided to exclude these data.

We assumed that species-specific occupancy probabilities varied across sites according to the relationships each species had to site-specific covariates. Using these covariates, we created 15 different candidate models falling into one of three categories: 1) Anthropogenic effects models, 2) Habitat models, 3) Coupled natural-human systems models. We did this to test whether anthropogenic variables, habitat related variables, or a combination of the two were best able to explain patterns in terrestrial mammal occupancy. The candidate models included:

Anthropogenic effects model:

$$H1)\;logit\left(\psi_{ij}\right)=\alpha_{i}*+\;{\alpha \;}_{i1}ROAD+\;{\alpha \;}_{i2}\text{FORESTLOSS}+\;{\alpha \;}_{i3}INDIG+\;{\alpha \;}_{i4}FIRE$$

$$H2)\;logit\left(\psi_{ij}\right)=\alpha_{i}*+{\alpha \;}_{i1}ROAD+\;{\alpha \;}_{i2}\text{FORESTLOSS}+\;{\alpha \;}_{i3}INDIG+\;{\alpha \;}_{i4}YEAR$$

Habitat Models:

$$E1)\;logit\left(\psi_{ij}\right)=\alpha_{i}*+{\alpha \;}_{i1}DTPA+\;{\alpha \;}_{i2}SWAMP+\;{\alpha \;}_{i3}FOREST+\;{\alpha \;}_{i4}YEAR$$

$$E2)\;logit\left(\psi_{ij}\right)=\alpha_{i}*+{\alpha \;}_{i1}DTPA+\;{\alpha \;}_{i2}SWAMP+\;{\alpha \;}_{i3}FOREST$$

Coupled Natural and Human Systems Models:

$$\mathrm{CA}1)\;logit\left(\psi_{ij}\right)=\alpha_{i}*+{\alpha \;}_{i1}DTPA+\;{\alpha \;}_{i2}\text{FORESTLOSS}+\;{\alpha \;}_{i3}SWAMP+\;{\alpha \;}_{i4}FIRE$$

$$\mathrm{CA}2)\;logit\left(\psi_{ij}\right)\;=\;\alpha_{i}*+\;{\alpha \;}_{i1}DTPA+\;{\alpha \;}_{i2}INDIG+\;{\alpha \;}_{i3}SWAMP+\;{\alpha \;}_{i4}FIRE$$

$$\mathrm{CA}3)\;logit\left(\psi_{ij}\right)\;=\;\alpha_{i}*+\;{\alpha \;}_{i1}SWAMP+\;{\alpha \;}_{i2}\text{FORESTLOSS}+\;{\alpha \;}_{i3}INDIG+\;{\alpha \;}_{i4}FOREST$$

$$\mathrm{CA}4)\;logit\left(\psi_{ij}\right)\;=\;\alpha_{i}*+\;{\alpha \;}_{i1}\text{FORESTLOSS}+\;{\alpha \;}_{i2}SWAMP+\;{\alpha \;}_{i3}FOREST+\;{\alpha \;}_{i4}IM$$

$$\mathrm{CM})\;logit\left(\psi_{ij}\right)\;=\;\alpha_{i}*+\;{\alpha \;}_{i1}SWAMP+\;{\alpha \;}_{i2}FOREST+\;{\alpha \;}_{i3}\text{FORESTLOSS}+\;{\alpha \;}_{i4}FIRE$$

$$\mathrm{CI})\;logit\left(\psi_{ij}\right)\;=\;\alpha_{i}*+\;{\alpha \;}_{i1}SWAMP+\;{\alpha \;}_{i2}FOREST+\;{\alpha \;}_{i3}INDIG+\;{\alpha \;}_{i4}FIRE$$

$$\mathrm{CH}1)\;logit\left(\psi_{ij}\right)=\alpha_{i}*+\;{\alpha \;}_{i1}DTPA+\;{\alpha \;}_{i2}SWAMP+\;{\alpha \;}_{i3}FOREST+\;{\alpha \;}_{i4}ROAD$$

$$\mathrm{CH}2)\;logit\left(\psi_{ij}\right)\;=\;\alpha_{i}*+\;{\alpha \;}_{i1}DTPA+\;{\alpha \;}_{i2}SWAMP+\;{\alpha \;}_{i3}\text{FORESTLOSS}+\;{\alpha \;}_{i4}ROAD$$

$$\mathrm{CH}3)\;logit\left(\psi_{ij}\right)\;=\;\alpha_{i}*+\;{\alpha \;}_{i1}SWAMP+\;{\alpha \;}_{i2}FOREST+\;{\alpha \;}_{i3}ROAD+\;{\alpha \;}_{i4}\text{FORESTLOSS}$$

$$\mathrm{CH}4)\;logit\left(\psi_{ij}\right)=\alpha_{i}*+{\alpha \;}_{i1}SWAMP+\;{\alpha \;}_{i2}FIRE+\;{\alpha \;}_{i3}ROAD+\;{\alpha \;}_{i4}\text{FORESTLOSS}$$

$$\mathrm{CH}5)\;logit\left(\psi_{ij}\right)=\alpha_{i}*+{\alpha \;}_{i1}SWAMP+\;{\alpha \;}_{i2}FOREST+\;{\alpha \;}_{i3}ROAD+\;{\alpha \;}_{i4}INDIG$$

We estimated the parameters in a Bayesian mode of inference in WinBUGS [23] through the R2WinBUGS package [24] in the program R using non-informative prior distributions. All covariates were centered and scaled with the exception of IM and YEAR. We used three chains to evaluate results of the model, running 25,000 iterations after a 5,000 burn-in. As suggested by Link & Eaton [25], we retained all data from simulations rather than setting a thinning rate. To assess for convergence we first reviewed the trace plots of the posterior distributions for irregularity and then assessed the potential scale reduction factor [26]. A potential scale reduction factor close to one indicates that the three simulations approach the target distribution [26]. When trace plots indicated convergence and a model’s scale reduction factor for all parameters was <1.1, we assumed the model had converged.

We ran models and assessed model fit using the model’s Deviance Information Criterion (DIC) value. Based on [27], we consider that a model has support if it is within 7 DIC units of the model with the lowest DIC value.

After our initial round of modeling, we created a binary covariate representing sampling methodology (METHOD). To assess if our study species’ detection probabilities varied according to sampling methodology we re-ran our highest ranking models with the detection probability model defined as:

$$logit\left(p_{ij}\right)=\alpha_{i}+\;+\;{\alpha \;}_{i1}METHOD$$

## Results

We collected camera-trapping data from a total of 122 independent sites in 2010–2012 and 2014. There was partial temporal replication across all years, such that when stacked we analyzed a total of 207 unique year/site combinations. Due to some camera malfunction, we were able to use the complete 44 trap nights of data from only 165 cameras, 33 trap nights of data from 21 cameras, 22 trap nights of data from 16 cameras, and 11 trap nights of data from 5 cameras. We thus used data from a total of 8,371 camera trap nights. Across all years our cameras collected a total of 1,083 unique detections of our eight study species. Our cameras collected multiple important records for Nicaragua, including giant anteater (*Myrmecophaga tridactyla*) photos in the Indio-Maíz and Wawashang Reserves, and a 2014 male jaguar photo from Bangkukuk, the community that, according to publications describing the proposed canal infrastructure, will be displaced by the canal’s Caribbean Coast deep water port and replaced with a dredge fill zone [28].

Trace plots and potential scale reduction factors suggest convergence for all models. The model with the lowest DIC and thus the highest-ranking model was an anthropogenic effects model, CA1 (DIC = 5095.9). All other models were >7 ΔDIC units from the highest ranking model and were not used for subsequent mapping.

It is important to note that our supported model does not include YEAR, which justifies our decision to stack data across years. Furthermore, in our two models that include year, H2 and E1, the only species for which we have evidence of a relationship between occupancy and YEAR is white-tailed deer (-). Despite the fact that neither of these models was <7 ΔDIC units from the highest-ranking model, we do not discuss white-tailed deer occupancy in great detail partially for this reason.

Also important to note is that we have no evidence of a relationship between METHOD and any species’ detection probability. Models with the METHOD covariate for detection had lower DIC values than the same model without the METHOD covariate. Results from CA1 with METHOD added can be reviewed in S1 Table.

**Fig 2:**
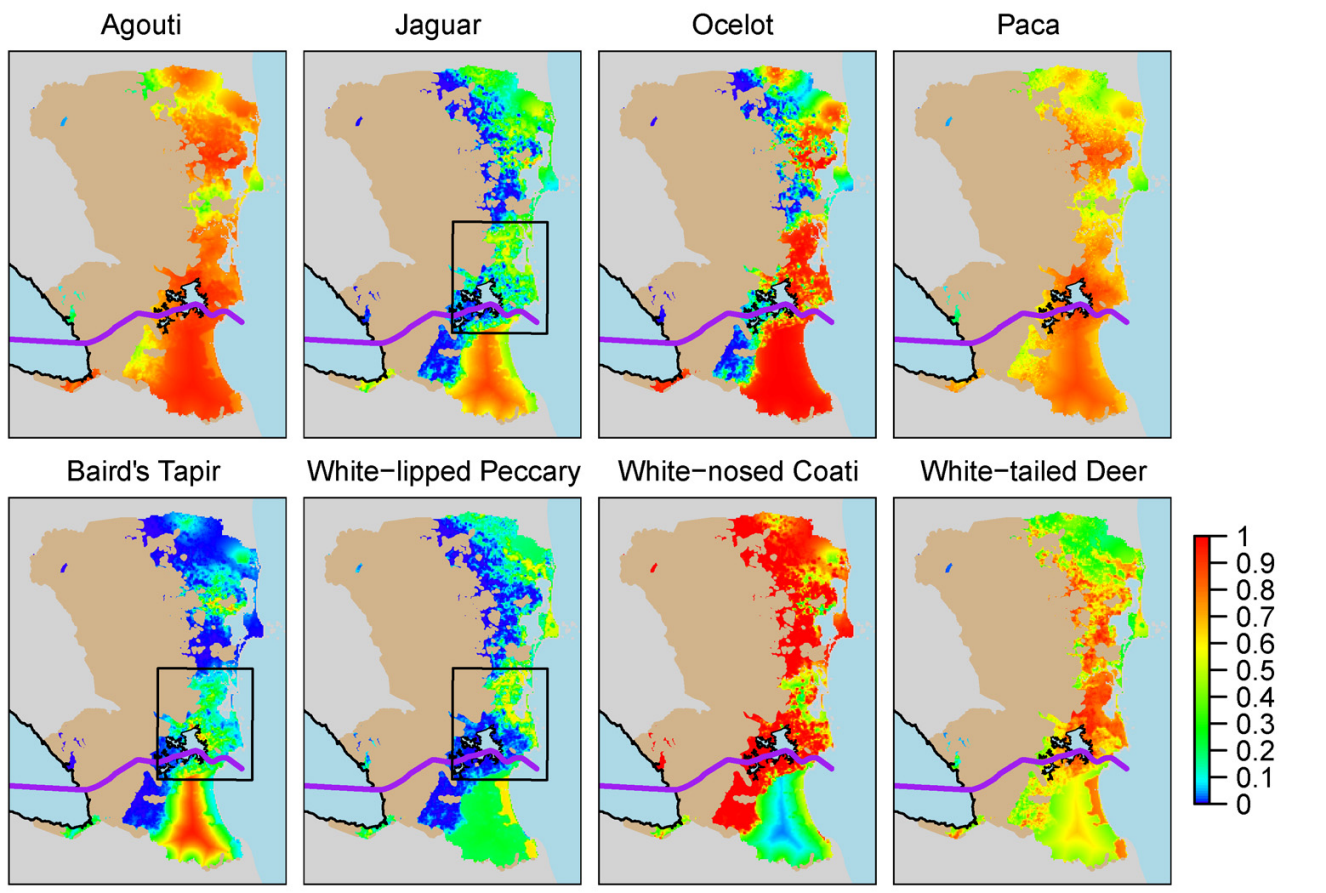
Occupancy maps for all species (model CA1). Black rectangle indicates extent of close up map (Fig 3), light brown area indicates agricultural land cover used as a mask.

We used the coefficients from model CA1 to create species-specific occupancy maps that enabled us to identify important core areas of habitat for all of our study species across the study area (**Fig 2**). Due to the fact that the forest loss layer included in model CA1 only accounts for the years 2000–2013, this layer does not appropriately account for regions that were deforested before 2000. Thus we masked our area of inference to exclude from our map all areas that were classified as “Agriculture” in Vreugdenhil et al. [20], which was generated using field data from 1999–2001.

In CA1, the covariates differ in their effect size and in their relationship to mammal occupancy. We consider that when the 95% Credible Interval does not overlap zero, there is evidence of a relationship [15]. We have consistent evidence of a strong relationship between jaguar, white-lipped peccary, and ocelot occupancy and our covariate for forest loss (-) (**Table 1**). We also have evidence of a relationship between white-lipped peccary and white-tailed deer occupancy and the area of wetlands around the camera site (+). Our models suggest that Baird’s tapirs have a strong relationship with distance from protected areas (-). We have evidence that Central American agouti (-) and ocelot (-) occupancy also have the same relationship with increasing distance from protected area. White-nosed coatis differ from most other species in their relationship with forest loss (+) and distance to protected areas (+). The results of models >7 ΔDIC units from the highest-ranking model can be reviewed in **S1 Table**.

**Table 1:** DIC values and coefficients for all species for model CA1. Bold type coefficients with an asterisk indicate zero is not included in the 95% credible interval. WLP = white-lipped peccaries, WNCT = white-nosed coatis, WTDR = white-tailed deer. DTPA = distance to protected area, FIRE = Number of fires within 1 km buffer of camera site, FORESTLOSS = Forest loss between 2000–2014 within a 1 km buffer around camera site, SWAMP = Area of swamps within 1 km buffer around camera site.

**Model Coefficients**				
Species	DTPA	FIRE	FORESTLOSS	SWAMP
**Agouti**	-0.469*	-0.028	-0.128	-0.229
**Baird's Tapir**	-1.505*	-0.674	-0.830	-0.437
**Jaguar**	-0.654	-0.317	-1.327*	-0.203
**Ocelot**	-1.549*	0.255	-1.225*	0.099
**Paca**	-0.392	-0.397	0.097	-0.095
**WLP**	-0.103	-1.002	-1.381*	0.643*
**WNCT**	1.123*	0.267	2.305*	0.179
**WTDR**	-0.375	0.475	0.316	0.567*

**Fig 3:**
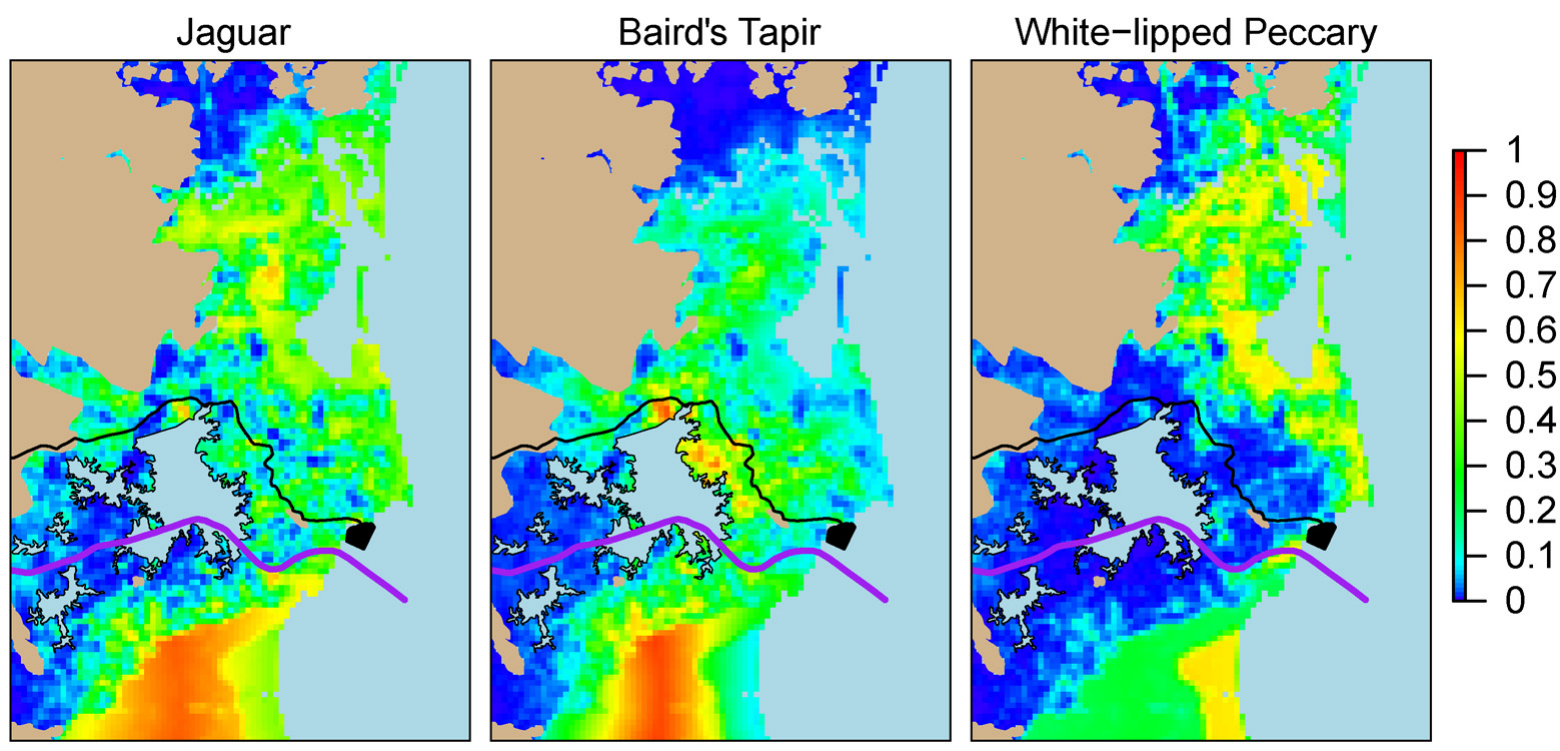
Close up of occupancy maps for rare species (model CA1), with planned infrastructure for the canal, including proposed road and dredge fill area (black) [10,28,29].

We digitized proposed canal infrastructure within our area of inference and placed it over occupancy maps for our three rare species: white-lipped peccaries, jaguars, and Baird’s tapirs (**Fig 3**) [10,28,29].

## Discussion/Conclusion

### Model Results

Our modeling results gave us insight on two general topics: a) the state of core areas for our study species in the southern Caribbean region of Nicaragua and b) the potential effects the proposed Nicaragua canal might have on the occupancy of our study species in the Caribbean region.

#### Core areas

Importantly, our results indicate that the entirety of the study area retains areas of high-predicted occupancy for at least one of our study species. Even in areas with higher forest loss, our generalist species, Central American agoutis; our two species expected to thrive in secondary habitat, white tailed deer and white-nosed coati; and lowland pacas, one of our species expected to be moderately sensitive to human encroachment and forest loss, have high-predicted occupancy. This indicates that even in relatively deforested landscapes in our study area substantial wildlife habitat remains, and suggests that our more common species thrive throughout most of our study area. This alone makes our study area significant for mammal conservation in Central America.

For our other species, ocelots and our three rare species, we conclude there are three areas with the potential to function as independent core areas, a term we interpret as a defined area of critical habitat: 1) The Indio-Maíz Biological Reserve, 2) The northern Cerro Wawashan Nature Reserve/Karawala Region, and 3) The Northern Cerro Silva Nature Reserve/Bluefields Region. While our data do not allow us to refute that gene flow exists between these core areas, we treat them as independent areas of critical habitat for two reasons: 1) because they are, in most cases, separated by areas of relatively lower predicted occupancy; and 2) each has a unique conservation status and conservation outlook, which makes discussing them separately more effective from a conservation and management standpoint.

In our study area, the Indio-Maíz Reserve is the largest block of contiguous habitat with high-predicted occupancy probabilities for most of our species. This is especially the case for jaguars, the globally endangered Baird’s tapir, and white-lipped peccaries. We conclude that Indio-Maíz is the only region in our study area that definitively functions as a core area for our rare study species. The agricultural frontier and the associated *Mestizo* hunters that invade portions of the reserve to hunt and extract timber and non-timber forest products have not yet converted Indio-Maíz into an empty forest. Indio-Maíz should still be considered a global priority for conservation in Central America and efforts should be made to ensure its protection in the coming years. Our preliminary data from a separate monitoring project indicate that Indio-Maíz is experiencing an increasingly massive invasion from cattle ranching *Mestizos*, which makes it particularly critical to carry out such conservation efforts now.

The other two potential core areas are smaller in size and have lower average predicted occupancy rates than Indio-Maíz such that we cannot confidently conclude that they function as core areas for our three rare species. Yet their size makes them significant conservation units that we believe could function as core areas under appropriate conservation and management programs. The first of these smaller potential core areas is the northern Cerro Wawashang Nature Reserve/Karawala region. Perhaps unexpectedly, the only protected area in the vicinity of Karawala is the roughly 1,000 hectare Llanos de Karawala Reserve, comprised entirely of pine savannah. Despite this, most of our study species, including jaguars, white-lipped peccaries, and ocelots, have relatively high predicted occupancy in the forests on the outskirts of this small protected area. Indeed, the broad-leafed forest and wetlands surrounding these pine savannahs are entirely unprotected by legislation, but our results suggest they are fairly well conserved by the local Ulwa indigenous people that reside in and use the ecosystems in and around Karawala. We posit that it is worth evaluating whether or not more formal national protection of this area would help the local indigenous people conserve their lands. Also of note for this region is that while the Cerro Wawashang Nature Reserve has been severely degraded in recent years [3], our results for jaguars, Baird’s tapirs, and a few other species suggest that there remains some suitable habitat within and surrounding the reserve, including: 1) in the far north of the reserve, 2) in the wetlands in the southeastern corner of the reserve, and 3) in the mangrove swamps to the east of the reserve that border several coastal communities where we occasionally hear reports of human-jaguar conflict. This means that for our rare species assemblage, efforts to restore lost habitat and reforest portions in the Cerro Wawashang Nature Reserve may allow them to repopulate the areas that Petracca et al. [3] indicate are currently unsuitable for jaguars.

The Northern Cerro Silva and Bluefields Region is the second of these smaller potential core areas. The size and significance of this area varies across our rare species; for ocelots this region is clearly connected by habitat with high predicted occupancy to Indio-Maíz. Unexpectedly, for most species, including jaguars and white-lipped peccaries, the most important habitat in this potential core area lies in the large system of wetlands behind Bluefields rather than in the Cerro Silva and Punta Gorda Nature Reserves further south. This is likely due to the severe degradation of these two reserves in recent years [4], but also probably reflects the relative inaccessibility of the swamps in and around Bluefields, which are dominated by *Raphia taedigera* Mart. palms. Our results suggest that these wetlands should be considered for more formal national level protection.

In a general sense, due to the amount of habitat predicted to have high occupancy rates for our rare species, including jaguars and Baird’s tapirs, we conclude that the RACS and RSJ remain very significant for the conservation of large terrestrial mammals in the neotropics. Nonetheless, the relative unsuitability of the habitat in the western portions of our study area for our three rare species, and the fact that this result is driven by the high level of recent forest loss in this region, are troubling. More troubling is that our results and the forest loss layer itself indicate that forest loss in Nicaragua has been extremely high over the past decade both inside and outside of protected areas [4]. Indeed, while the Southern Caribbean region of Nicaragua still holds fairly large areas of suitable habitat for terrestrial mammals, including rare, larger mammal species, both anecdotal data and our analyses indicate that it is under a growing threat from anthropogenic threats, most notably forest loss and an encroaching agricultural frontier. Our modeling provides evidence that these agricultural frontier effects have a much larger impact on terrestrial mammal occupancy in our study area than any natural variation in local ecosystems. The effects of the agricultural frontier are especially severe in the Punta Gorda Nature Reserve along the route of the proposed canal and in the Kukra Hill Region, which is the site of a large oil palm plantation. To ensure the conservation of our three rare study species, it is essential to halt the agricultural frontier specifically and bolster conservation efforts generally in: 1) the three areas that our results suggest have potential to function as important core blocks of habitat to prevent their degradation in the case of Indio-Maíz and to prevent them from functioning as ecological sinks for our study species in the case of the two smaller core areas; and 2) in the marginal habitat between the three potential core areas to help ensure that genetic connectivity is not lost between them.

#### b) Proposed interoceanic canal

It is important to note that according to the ecosystem layer we used to clip our study area, the majority of the terrestrial portion of the proposed Canal Route traverses agricultural habitat that was permanently deforested pre-2000 [20]. Cattle ranching dominates these landscapes. While our five study species that either thrive in secondary habitat or are less sensitive to human encroachment may be able to persist in areas dominated by cattle ranching, especially if ranchers conserve some patches of natural habitat and limit hunting, the same is not true for our more sensitive, rare species that require large patches of contiguous habitat to persist. Thus, we assume that none of the areas classified as agriculture before 2000 retain habitat occupied by jaguars, white-lipped peccaries, and tapirs. Indeed, the only area where we would expect to find these three species is in the Caribbean region east of these cattle ranching landscapes: our area of inference in our mapped occupancy projections (i.e. **Fig 3**). Given this scenario, and that we have a large sample size from this Caribbean Region, we contend that we are in a position to make an informed evaluation of the potential effects of the Nicaraguan Canal project on the occupancy rates of our three rare terrestrial mammal study species.

The proposed canal route does not traverse expansive areas of high-predicted occupancy for our three rare species. In fact, when Lake Atlanta is overlaid on top of the relative occupancy maps for these species, it becomes clear that along the proposed canal route, the only apparently suitable habitat that will remain above water for jaguars, white-lipped peccaries, and Baird’s tapirs is located in a relatively thin strip of patchy forests between the eastern edge of this proposed artificial lake and the coast (**Fig 3**). When all three rare species maps are considered jointly, the thin strip of forest along the Caribbean beach jumps out as perhaps the conservation priority and potentially an important dispersal corridor, especially for jaguars and white-lipped peccaries. This is notable because the ecosystems close to the coast is also the area that will have the highest density of canal infrastructure, including the canal itself, an access road, and a dredge fill area all in a relatively small area.Our results do not allow us to draw definitive conclusions about genetic connectivity in the proposed Canal Zone. Nonetheless, as discussed above, we would not expect these three rare species to occupy the agricultural landscapes along the segments of the proposed canal route that lie outside of the area of inference in our maps. Furthermore, of all the ecosystems along the proposed canal route, those that fall within our study area are the closest to the Indio-Maíz core area and the Northern Cerro Silva Nature Reserve/Bluefields potential core area. Due to this, we contend that even in the context of the planned expropriation of land from *Mestizo* cattle ranchers to make way for canal development, there will only be a chance of conserving genetic connectivity for jaguar, Baird’s tapirs, and white-lipped peccaries if the small strip of forest from the eastern edge of the proposed Lake Atlanta to the Caribbean coast is treated as a conservation priority during and after canal construction.

Based on this, we recommend adjusting the canal design and plans for the management of the canal and related infrastructure to avoid developing the area from the eastern edge of Lake Atlanta to the forests along the Caribbean coast beaches. These adjustments should be made in a way that specifically accommodates and increases the probability that large mammals can disperse across and survive in the vicinity of the proposed Canal Zone and could perhaps be accomplished in a number of ways, including:

1. Moving the location or adjusting the size of Lake Atlanta to minimize the flooding of areas where our three rare species have relatively high predicted occupancy;
2. Building small, forested islands along the Eastern edge of Lake Atlanta that species could use as small refuges as they disperse along the edge of the artificial lake;
3. Maximizing the protection of the canal’s riparian forests between Lake Atlanta and the Caribbean Coast and bolstering the protection of those core areas closest to the canal (i.e. Indio-Maíz and the Northern Cerro Silva/Bluefields region);
4. Including specific mechanisms, structures or management measures for the canal in the Caribbean region between Lake Atlanta and the coast that might allow large mammals to cross the canal or persist in the vicinity of the canal (i.e. avoid canal traffic or lights during those hours when wildlife are most likely to pass);
5. Including specific mechanisms, structures or management measures for access roads or ancillary development projects that would increase the probability that large mammals would cross or persist in the vicinity of the infrastructure. This is especially necessary in the case of the planned access highway that would bisect the conservation priority forests between the eastern edge of Lake Atlanta and the Caribbean coast (**Fig 3**).

In our study area, the habitat along the proposed Canal Zone is one of the areas of lowest mean predicted occupancy for our three rare species. Due to this, and given plans to build a high density of canal related infrastructure in the few areas where they do have relatively high predicted occupancy within the proposed canal route (**Fig 3**), we consider that in the absence of advanced conservation planning and substantial investments in specific, targeted mitigation efforts, it is likely that this project will extirpate jaguars, white-lipped peccaries, and Baird’s tapirs from the canal zone. While some express optimism [29], to date we have seen no published recommendations or plans indicating that the canal company or the Nicaraguan government are prepared to undertake the conservation actions required to avoid this fate for these rare and nationally imperiled species [16, 30].

## Supporting Information

S1 Table Coefficients and DIC for all models >7 ΔDIC units from the highest ranking model including the CA1 METHODS model. (CSV)

## References

[1] Hansen MC, Potapov PV, Moore R, Hancher M, Turubanova SA, Tyukavina A, et al High-resolution global maps of 21st-century forest cover change. Science. 2013;342(6160):850–853. doi: 10.1126/science.1244693 24233722 10.1126/science.1244693

[2] Buck Holland M. Mesoamerican Biological Corridor In: Hilley JA, Chester CC, Cross MS, editors. Climate and Conservation: Landscape and Seascape Science, Planning, and Action. Island Press; 2012 p.56–66.

[3] Petracca LS, Hernández-Potosme S, Obando-Sampson L, Salom-Pérez R, Quigley H, Robinson HS. Agricultural encroachment and lack of enforcement threaten connectivity of range-wide jaguar (Panthera onca) corridor. J Nat Conserv. 2014;22(5):436–444.

[4] Watsa ME. 'Natural Reserves' no more: illegal colonists deforest huge portions of Nicaraguan protected areas. Published August 13, 2014. Available: http://news.mongabay.com/2014/0813-gfrn-watsa-silva-gorda.html

[5] Schank C, Mendoza E, Garcia Vettorazzi MJ, Cove MV, Jordan CA, O’Farril G, et al Integrating current range-wide occurrence data with species distribution models to map potential distribution of Baird’s tapir. Tapir Conser. 2015;24(33):15–25.

[6] Jordan CA, Urquhart GR. Baird’s tapirs (*Tapirus bairdii*) in Nicaragua. Tapir Conserv. 2013;22(30):14–21.

[7] Rabinowitz A, Zeller K. A range-wide model of landscape connectivity and conservation for the jaguar, *Panthera onca*. Biol Conserv. 2010;143:939–945.

[8] Jordan CA, Galeano MR, Alonzo AS. La Cacería Historica de Tapires Centroamericanos (Tapirus bairdii) en la RAAS, Nicaragua. Estud Ambient. 2014;1(1):73–87.

[9] Huete-Pérez JA, Alvarez PJJ, Schnoor JL, Rittmann BE, Clayton A, Acosta ML, et al Scientists raise alarms about fast tracking of interoceanic canal through Nicaragua. Envir Sci Tech. 2015;49(7):3989–3996. 10.1021/acs.est.5b00215 25730497

[10] Hong Kong Nicaraguan Canal Development Group (HKND). Nicaragua Canal Project Overview. 2014; Available: http://hknd-group.com/upload/pdf/20141221/Nicaragua_Canal_Project_Overview_ENG_20141216.pdf

[11] Christie P, Bradford D, Garth R, Gonzalez B, Hostetler M, Morales O, et al Taking care of what we have: Participatory Natural Resource Management on the Caribbean Coast of Nicaragua. IDRC/CIDCA; 2000.

[12] Jordan CA. The dynamics of wildlife and environmental knowledge in a bioculturally diverse coupled natural and human system in the Caribbean region of Nicaragua [dissertation]. East Lansing (MI): Michigan State University; 2015.

[13] Royle JA, Dorazio RM. Hierarchical Modeling and Inference in Ecology. Boston (MA): Academic Press; 2008.

[14] Zipkin E, Royle JA. A hierarchical approach to multi-species inference. Available: http://www.mbr-pwrc.usgs.gov/site/communitymodeling/home/

[15] Linden DW, Roloff GJ, Kroll AJ. Conserving avian richness through structure retention in managed forests of the Pacific Northwest, USA. Forest Ecol Manag. 2012;284:174–184.

[16] Jovenes Ambientalistas. Lista roja de especies en alto riesgo. Managua: 2013. Available: http://www.bibliotecavirtualelmalinche.info/images/documentos/BVEM-0005.pdf

[17] IUCN. Tayassu pecari. The IUCN Red List of Threatened Species. 2013; Available: http://www.iucnredlist.org/details/41778/0

[18] OpenStreetMap contributors. Downloaded July 2015. Available: https://www.openstreetmap.org/.

[19] IUCN and UNEP-WCMC (2015), The World Database on Protected Areas (WDPA). Cambridge, UK: UNEP-WCMC Available: http://www.protectedplanet.net. Accessed July 2015.

[20] Vreugdenhil D, Meerman J, Meyrat A, Gómez LD, Graham DJ. 2002 Map of the Ecosystems of Central America: Final Report. World Bank, Washington, D.C. Available: http://documents.worldbank.org/curated/en/2002/01/6596824/map-ecosystems-central-america-vol-1-2-main-report

[21] Centre for Socio-Environmental Information (CISA). University of the Autonomous Regions of the Caribbean Coast of Nicaragua (URACCAN).

[22] NASA. This data set was produced by the University of Maryland and provided by NASA FIRMS operated by NASA/GSFC/ESDIS with funding provided by NASA/HQ. 2015. Available: https://earthdata.nasa.gov/active-fire-data#tab-content-6

[23] Spiegelhalter DJ, Thomas A, Best NG, Lunn D. WinBUGS Version 1.4 User Manual. MRC Biostatistics Unit: Cambridge (UK); 2003.

[24] Sturtz S, Ligges U, Gelman A. R2WinBUGS: a package for running WinBUGS from R. J Stat Softw. 2005;12:1–16.

[25] Link WA, Eaton MJ. On thinning of chains in MCMC. Methods in Ecology and Evolution. 2012;3(1):112–115.

[26] Brooks SP, Gelman A. General methods for monitoring convergence of iterative simulations. J Comput Graph Stat. 1998;7(4):434–455.

[27] Spiegelhalter DJ, Best NG, Carlin BP, Van Der Linde A. Bayesian measures of model complexity and fit. J Roy Stat Soc B. 2002;64(4):583–639.

[28] Kilpatrick K. Canal ‘Will Destroy We’: Bangkukuk residents worry canal will be final blow to their language and traditional culture. Published April 29, 2015. Available: http://projects.aljazeera.com/2015/04/nicaragua-canal/displaced.html

[29] Condit R. Extracting environmental benefits from a new canal in Nicaragua: lessons from Panama. PLoS Biol 13(7): e1002208 doi: 10.1371/journal.pbio.1002208 26214182 10.1371/journal.pbio.1002208 PMC4516262

[30] Hong Kong Nicaraguan Canal Development Group (HKND). Environmental and Social Impact Assessment; Available: http://hknd-group.com/portal.php?mod=view&aid=293

